# Clinical and imaging features of melanotic neuro-ectodermal tumor of infancy of the maxillary bone: report of four cases and review of the literature

**DOI:** 10.1007/s11282-022-00638-7

**Published:** 2022-07-25

**Authors:** Yujiao Guo, Ying Liu, Xingang Wang, Gang Li, Guoxia Yu

**Affiliations:** 1 Department of Stomatology, Beijing Children’s Hospital, Capital Medical University, National Center for Children’s Health, Beijing, 100045 China; 2 Department of Oral and Maxillofacial Radiology, Peking University School and Hospital of Stomatology, Beijing, 100081 China

**Keywords:** Melanotic neuro-ectodermal tumor of infancy, Maxillary bone tumor, Imaging features, Pediatrics, Clinical manifestations

## Abstract

Melanotic neuro-ectodermal tumor of infancy (MNTI) is an extremely rare tumor. The purpose of this study was to describe the imaging features of maxillary bone MNTIs and introduce the key points for clinical diagnosis of MNTI. We retrospectively reviewed four patients with histology-proven MNTIs arising from the maxillary bone. All patients underwent ultrasonic inspections, CT and/or MR scanning. Combined with previously literature, the imaging features were comprehensively evaluated and analyzed. All MNTIs showed a firm, non-ulcerated rapidly-growing soft-tissue swelling with pigmented (blue-colored or black-colored or gray-colored) mucosa. The onset ages were younger than 6 month-old. CT images showed osteolytic or expansive bone destruction of the involved maxillae, fragmentary cortical bone, “free-floating” tooth germs, with or without spiculated/sunburst periosteal reaction. The tumor appeared lightly hyper-intense on T2-weighted sequences, while isointense or lightly hypo-intense or lightly hyper-intense signal on T1-weighted sequences. Enhanced images all displayed heterogeneous enhancement. No metastasis features of lymph nodes or abdominal organs were demonstrated by cervical and abdominal ultrasonic inspections. As a conclusion, accurate recognition of the imaging features of MNTI combined with history and clinical manifestations (early infancy, painless, firm, pigmented mucosa, non-ulcerating lesion) can provide clues for diagnosis of this rare entity.

## Introduction

Melanotic neuroectodermal tumor of infancy (MNTI) is an extremely rare and rapidly growing neoplasm which originates from neural crest. The tumor usually develops in the head and neck regions of early infancy, particularly in the maxilla, followed by the skull, mandible and brain [1–3]. MNTIs are generally classified as benign but local-aggressive neoplasms [4, 5]. It is essentially a rapid-growing painless tumor causing facial malformation, while the mucosa surface is intact, pigmented and non-ulcerated [4, 6]. To the best of our knowledge, there have been few studies focusing on the imaging findings of MNTI to date, and most of the studies were MNTIs from the skull [7–12]. During the past five years, four cases of MNTI occurring in maxillary bone have been confirmed by histopathology in our hospital. The imaging findings of these four MNTIs were retrospectively reviewed and, combined with the literature, the value of using CT, MR and ultrasound imaging to diagnose, treat and follow up patients with MNTIs in the maxillary bone was also discussed. Imaging examination strategies for these patients were explored too.

## Case reports

The study was approved by the institutional review board. We retrospectively reviewed the imaging findings of four patients with histology-proven MNTIs over a 5 year period (July 2016–June 2021) by searching the clinical records. All four patients underwent surgical treatment. The clinical presentations, surgical findings and histological diagnosis were extracted from the medical records.

The four patients all presented to the department of oral and maxillofacial surgery and underwent CT examination. Images were acquired in both the axial and coronal planes using Revolution CT, GE Healthcare. The imaging parameters were as follows: voltage 100 kV, current 200 mA, matrix 512 × 512, section thickness 0.625 mm. Examinations were performed from superciliary arch to the submandibular region. Images were reconstructed using both bone algorithm (window width 2000 HU at a window level of 600 HU) and soft-tissue algorithm (window with 350HU, window level 40HU). Case 1 and case 2 got enhanced CT check in which 2 ml of iodixanol injection (Visipaque, GE Healthcare Ireland Limited) per kilogram of body weight was applied via intravenous injection.

Case 3 and case 4 also underwent MR examination prior to surgery. All MRI was acquired on a 3-T scanner (Achieva, Philips Medical System, Best, the Netherlands), using a head coil. They underwent pre-enhanced T1- and T2-weighted scanning and case 3 also had post-enhanced T1-weighted images in the axial, coronal and sagittal planes. The imaging parameters were as follows: T1-weighted images: repetition time (TR) 500–600 ms, echo time (TE) 15–20 ms; T2-weighted images: TR 4000 ms, TE 80–100 ms, matrix 512 × 512, field of view 20 × 20 cm, and section thickness 2.5 mm. Rapid manual bolus intravenous injection (2 ml s^−1^) of 0.1 mmol of gadopentetate dimeglumine (MagnevistH; Schering AG, Berlin, Germany) per kilogram of body weight was administered.

In addition, cervical and abdominal ultrasonic inspections were also performed for screening metastasis features of lymph nodes or abdominal organs.

All the images were evaluated by three experienced oral and maxillofacial surgeons/radiologists and findings were reached by consensus.

Biopsy operations were scheduled for the four patients before resection operations. The final pathological diagnosis of MNTI by surgery was consistent with biopsy.

### Case 1

**Fig. 1:**
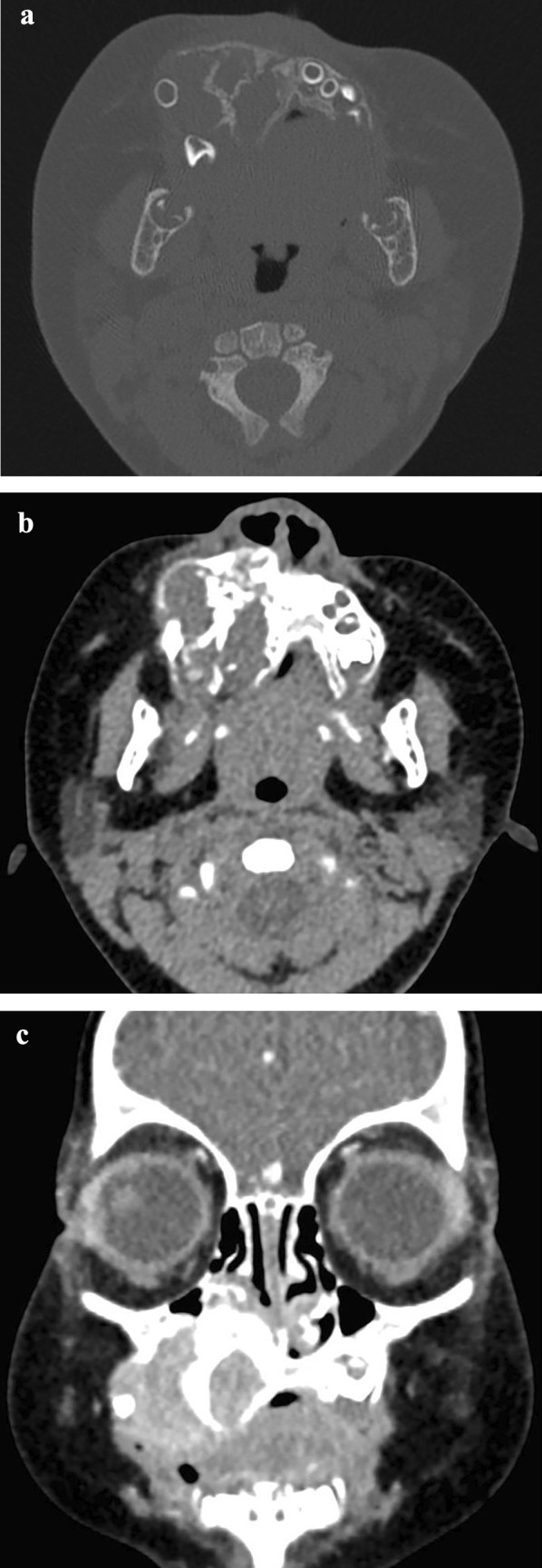
Case 1. **a**/**b** axial CT images demonstrated a multi-locular soft-tissue mass occupying the right maxillary sinus and leading to irregular bony destruction. Cortex expanded and lost continuity without periosteal or bony proliferation. Displaced tooth germ was noticed. **c** coronal contrast-enhanced CT showed heterogeneous enhancement of the lesion

A 6 month-old male presented with a 3 month history of painless swelling of the right maxillary region. Clinical examination showed a non-tender, non-ulcerated, blackish-grayish tumor causing craniofacial asymmetry. CT demonstrated a multi-locular soft-tissue mass occupying the right maxillary sinus and leading to irregular bony destruction. Cortex expanded and lost continuity without periosteal or bony proliferation. Several displaced tooth germs were noticed in the tumor. Contrast-enhanced CT showed heterogeneous enhancement of the lesion (Fig. 1). Diagnosis of MNTI was made by biopsy. The cervical and abdominal ultrasonic inspections showed no metastasis features of lymph nodes or abdominal organs. The patient was given surgery by enucleation under general anesthesia, and monitored every 6 months for 3.5 years, without evidence of recurrence.

### Case 2

**Fig. 2:**
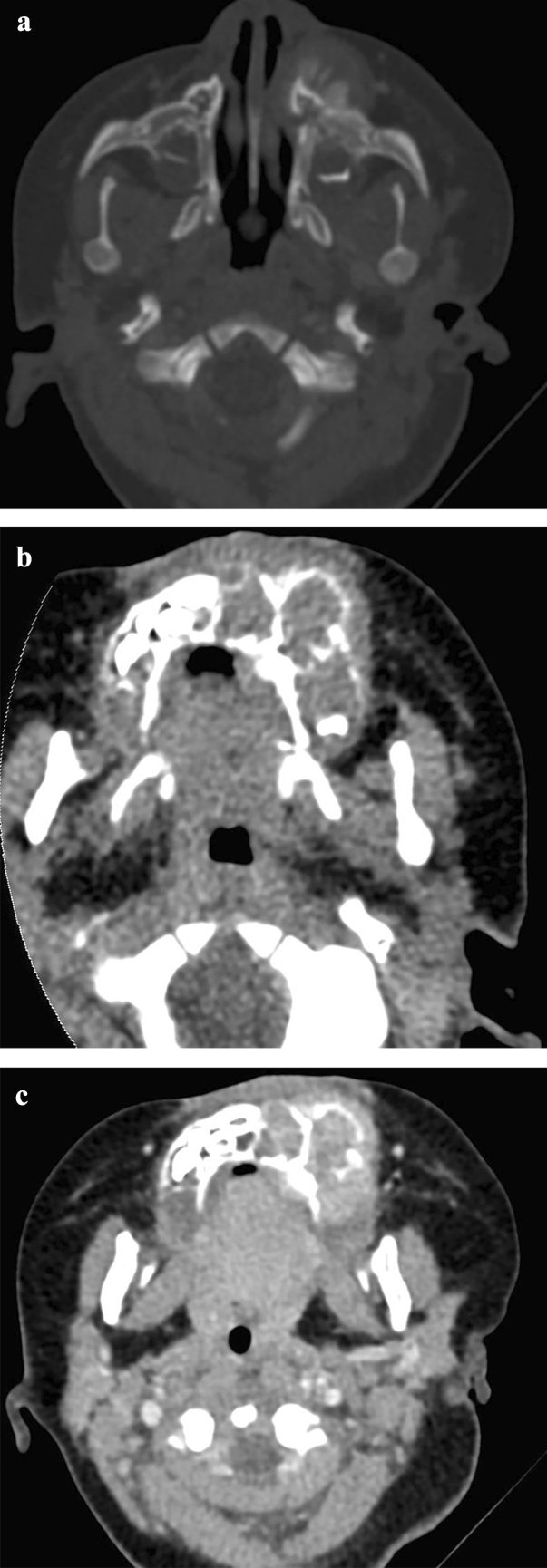
Case 2. **a**/**b** axial CT images revealed osteolytic and expansive bone destruction of left maxilla, involving all maxillary sinus walls. Spiculated/ sunburst periosteal reaction was obvious. Irregular and ill-defined soft-tissue mass in which tooth germs floated was noticed in the bone destruction area. **c** axial contrast-enhanced CT scans showed medium heterogeneous enhancement of the soft-tissue mass combining with liquefactive necrosis areas

A 1-month-old female was admitted owing to a rapidly growing tumor of left maxilla of 2 weeks’ duration. The patient had left nasal obstruction, proptosis, and facial malformation. Clinical examination revealed a large and firm mass from midline to left maxillary tuberosity. Mucosa surface of the tumor was intact with blueish-grayish color. CT revealed osteolytic and expansive bone destruction of left maxilla, involving all maxillary sinus walls and inferior orbital wall. Spiculated/sunburst periosteal reaction was obvious. Irregular and ill-defined soft-tissue mass in which tooth germs floated was noticed in the bone destruction area. Contrast-enhanced CT scans showed medium heterogeneous enhancement of the soft-tissue mass combining with liquefactive necrosis areas (Fig. 2). Diagnosis of MNTI was made by biopsy. No metastasis features of lymph nodes or abdominal organs were demonstrated by cervical and abdominal ultrasonic inspections. The patient also had eye examinations and fibro nasopharyngoscopy due to nasal obstruction and proptosis. Curettage was taken to treat the patient under general anesthesia, while 1 month later, a recurrent lesion was found in the left maxilla. Thus, a second enucleation surgery with subtotal resection of maxilla was undertaken. Regular follow-up examination 31 months after the second surgery revealed no evidence of recurrence.

### Case 3

**Fig. 3:**
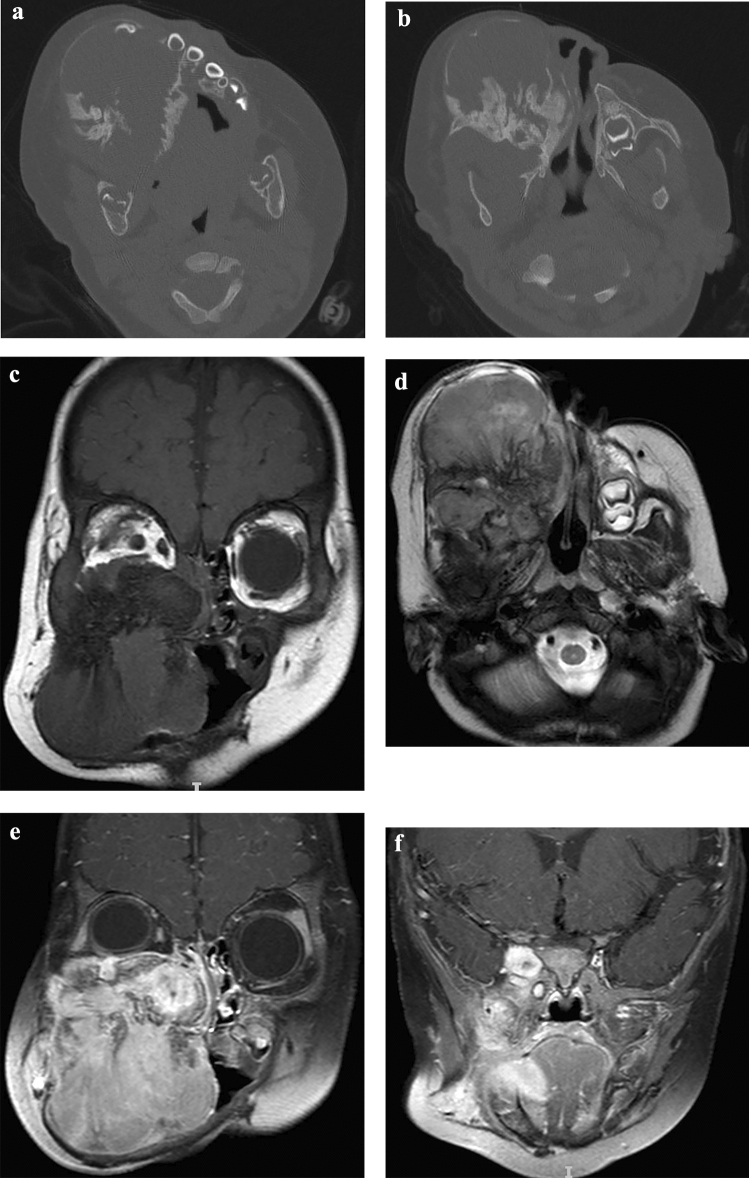
Case 3. **a**/**b** axial CT showed expansive tumor with obscure boundary, leading to extensive bone destruction, local spiculated periosteal reaction and fragmentary cortical bone. Several displaced/ “free-floating” teeth were distinct. **c** coronal T1 weighted image showed isointense or lightly hypo-intense signal. **d** axial T2 weighted image showed lightly hyper-intense signal. **e**/**f** coronal enhanced T1-weighted images showed intense but inhomogeneous enhancement following gadolinium injection. **c**/**d**/**e**/**f** Right maxilla, infratemporal fossa, pterygopalatine fossa, orbital apex and cavernous sinus were involved. Right orbit was smaller than the contralateral side causing by the compression of neoplasm. Nasal septum was also compressed tipping to left side

A 7 month-old male was referred with progressive right facial swelling for 3 months. The patient suffered from right nasal obstruction, feeding difficulty, ocular proptosis and facial deformity. Clinical examination showed giant (about 10 cm * 8 cm) and non-ulcerated invading right orbit, nasal cavity and whole right maxilla. Mucosal of the tumor was intact with grayish–whitish or grayish–blackish color. CT showed tissular expansive tumor with obscure boundary, leading to extensive bone destruction, local spiculated periosteal reaction and fragmentary cortical bone. Right maxilla, infratemporal fossa, pterygopalatine fossa, orbital apex and cavernous sinus were involved. Several displaced/ “free-floating” teeth were distinct. Right orbit was smaller than the contralateral side causing by the compression of neoplasm. Nasal septum was also compressed tipping to left side. On MRI, the tumor appeared isointense or lightly hypo-intense on T1-weighted sequences, lightly hyper-intense on T2-weighted sequences, and show intense but inhomogeneous enhancement following gadolinium injection. Involvement of right orbit, orbit apex area, cavernous sinus and right optic nerve was more clear on MRI than CT (Fig. 3). The cervical and abdominal ultrasonic examinations seemed quite necessary for this child, while no metastatic lymph nodes or abdominal organs were found. The lesion was excised by enucleation with subtotal resection of maxilla under general anesthesia. Clinical and imaging follow-up every 6 months for 26 months showed no evidence of recurrence.

### Case 4

**Fig. 4:**
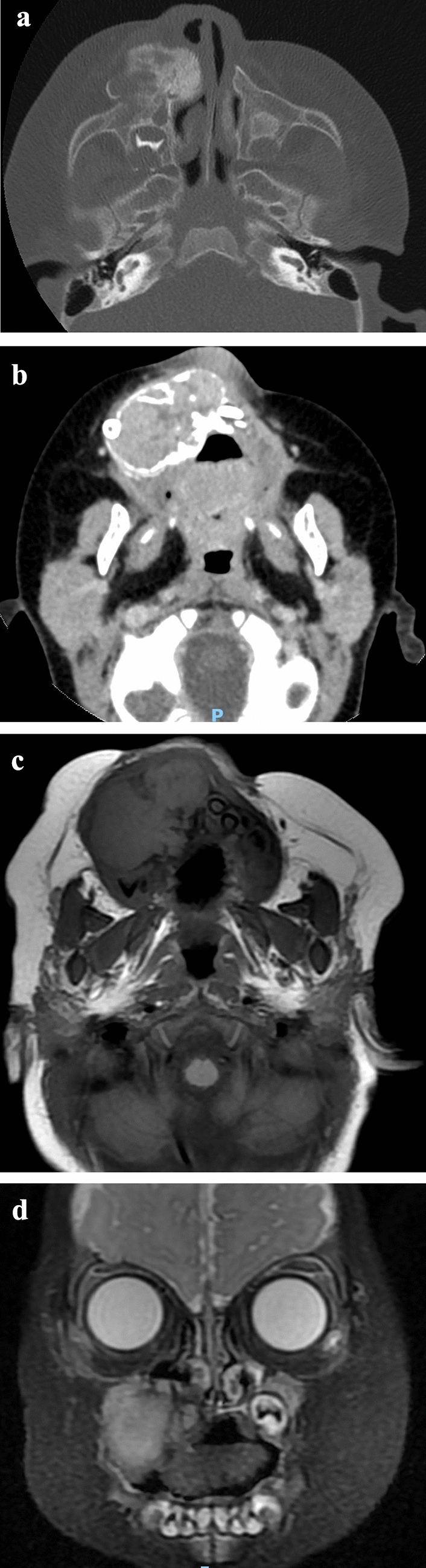
Case 4. **a**/**b** axial CT images showed irregular bone destruction and cortical bone expansion, with no signs of periosteal reaction. Teeth in the lesion were displaced. **c** axial T1 weighted image showed isointense or mild hyper-intense signal. **d** coronal T2 weighted also showed isointense or mild hyper-intense signal

A 5 month-old male was referred because of a rapidly growing tumor of the maxilla. The first symptoms of swelling in the right maxilla region had been noticed about 2 months before. Clinical examination revealed a firm, lobed, non-ulcerated, reddish-bluish tumor with intact mucosa. CT scans showed irregular bone destruction and cortical bone expansion, with no signs of periosteal reaction. Teeth in the lesion were displaced. On MRI, the mass was isointense or mild hyper-intense on both T1- and T2-weighted sequences with clear boundary (Fig. 4). Surgical enucleation of the tumor mass was planned under general anesthesia. During the 12 month follow-up period, there was no evidence of recurrence of the mass.

## Discussion

MNTI is generally found in children younger than 1 year of age, especially between 2 and 6 months of age [2, 13, 14]. Cases of prenatal diagnosis were quite rare [15]. The onset ages of our four cases were all younger than 6-month-old. Clinical presentations of the four cases were also in line with previous publications [4, 13, 16]. MNTI usually occurs in the head and neck region due to its origin from the neuro-ectoderm. Around 70% present in the maxilla, followed by the skull (11%) and mandible (6%) [2, 4, 5]. Patients typically present a firm, non-ulcerated rapidly growing soft-tissue swelling with pigmented (blue-colored or black-colored or grey-colored) mucosa, causing facial deformity, feeding difficulty, even dyspnea [4–6, 13, 17].

The clinical and imaging findings of previously published MNTIs of the maxillary bone in the literature are summarized in Table 1.

**Table 1:** melanotic neuro-ecto-dermal tumors of infancy of maxillary bone reported in the literature

Author	Sex (M/F)/ Age (m/y)	Symptoms and history	Tumor surface	Laterality	Imaging findings
Butt et al. [1]	F/2y	Deformity of the face for 1 year	NM	Left maxilla	A clearly delineated lesion occupying maxillary sinus (CT)
Kruse-Losler [2]	M/7 m	Swelling in the maxillary region for 2 months	Non-ulcerated, reddish bluish color	Right maxilla	a lobed extensive tumor with bone destruction, weak contrast enhancement, and displaced dental germs, and central hypo-dense areas (CT)
Higashi K et al. [5]	F/3 m	Rapidly growing tumor of maxilla invading lacrimal sac for 1 month	Reddish overlying mucosa	Left maxilla	Giant soft-tissue mass with bone destruction (CT)
	M/7 m	Gradually growing tumor for 2 months	NM	Median maxilla	Radiolucent lesion with clear margin (CT/MRI)
Chaudhary et al. [6]	18cases: 12 M, 6F Mean age 4.3 ± 2.1 m	Facial asymmetry or upper lip swelling or feeding difficulty	Smooth, thin, non-ulcerated mucosa with bluish back in color	13 cases in lateral maxillary alveolus; 2 cases in midline maxillary; 3 cases in alveolus and hard plate	Central area of radiolucency with sharp margins displacing the surrounding bone and tooth buds (CT) Hypo-intense mass with focal areas of hyper-intensity in T1W images and an isointense mass on T2W images (MRI) Ultrasound of the abdomen and whole body skeletal screening was normal (US)
Haque et al. [7]	M/1 m	Rapidly growing tumor for 2 weeks	Bluish, non-ulcerated mucosa	Left maxilla	Isointense soft-tissue mass (CT) Predominantly hypo-intense on both the T1- and T2- weighted sequences (MRI)
Nazira et al. [10]	M/2.5 m	Rapidly progressive swelling for a few weeks	Reddish-bluish overlying mucosa	Left maxilla	bilobular, expansile bone lesion with homogenous soft-tissue density content,and displaced dental germs. Moderate enhancement (CT) Hyper-intense signals on T1W images and mildly hyper-intense signals on T2W images, moderate enhancement (MRI)
Moreau et al. [14]	11cases, 1 in mandible, 10 in maxilla: Mean age 2.82 m (range: prenatal to 5 m)	NM	NM	NM	Tumor invasion of the orbit, cortical osteolysis, enhanced after injection(CT) In T1W sequences, hypo-intense in 2 cases, isointense in 6 cases, and hyper-intense in 2 cases, in T2W sequences, hypo-intense in 5 cases, hyper-intense in 6 cases. All enhanced after gadolinium injection (MRI)
Magliocca [17]	M/6 m	A mass for 2 months	Bluish color	Left anterior maxilla	Radiolucent lesion with expansile bone remodeling and displacement of regional tooth germs (CT)

Sex (M/F), sex (male/female); Age (m/y), age (months/years)

*NM* not mentioned; *CT* computed tomography; *MRI* magnetic resonance image; *T1W* T1-weighed; *T2W* T2-weighed; *US* ultrasound

The radiological appearance seen in our patients had similar features. CT can be used to describe bone change, tooth character and adjacent anatomical structures, as well as to define the mass for surgical approach [6]. In our patients, involved maxillae all presented osteolytic bone destruction, expansive bone destruction and fragmentary cortical bone. Case 2 and case 3 also showed spiculated/sunburst periosteal reaction, suggesting a rapid-growing or even malignant process [9]. Displaced or “free-floating” tooth germs can be seen in all the four patients. MRI can better illustrate the extent of the soft tissue component. Pigment melanin is paramagnetic material. Tumors which contain melanin usually display hyper-intense signal on the T1-weighted image, while hypo-intense or isointense signal on the T2-weighted image [10, 14, 18]. Actually, MR images of MNTI do not always fit this pattern, because of different melanin content and different bone destruction in the tumor [7, 8, 10]. Case 3 showed isointense or lightly hypo-intense signal on T1-weighted sequences, and lightly hyper-intense signal on T2-weighted sequences. While case 4 showed isointense or mild hyper-intense signals on both T1- and T2-weighted sequences. The MR signals of our cases were unlike a typical melanin signal, but signals of bone involvement, tumor necrosis region, inflammatory reaction around the tumor and so on [10, 14]. Enhanced CT/MR images in these four cases all displayed heterogeneous/ inhomogeneous enhancement, which was in line with previous literatures [7, 10].

As MNTIs in maxilla often invade adjacent orbit, nasal cavity even cranial base, ophthalmic testing, otolaryngological examination and neurosurgical examination shall be taken when necessary [19]. Although generally considered benign, the biologic behavior of MNTI is not fully understood, rapid growth rate, significant risk of local recurrence [14, 20, 21]. Local aggressive behavior, rapid expansible growth, recurrence and malignant transformation were sometimes reported in literature [4, 22–24]. Therefore, the cervical and abdominal ultrasonic inspections are also recommended to detect local and distant metastasis. No metastasis features of lymph nodes or abdominal organs were found in our four cases. After all these results of auxiliary examination taken into consideration, individualized treatment programs can be achieved probably.

In conclusion, MNTI is extremely rare, it can occasionally occur in the maxillary bone. As to radiologists, accurate recognition of imaging features of MNTI combined with history and clinical manifestations (early infancy, painless, firm, blue-colored or black-colored or grey-colored, non-ulcerating lesion) can provide clues for diagnosis for MNTI, although the final diagnosis was achieved by pathological diagnosis after surgery.
